# Acute effect of Ceylon cinnamon extract on postprandial glycemia: alpha-amylase inhibition, starch tolerance test in rats, and randomized crossover clinical trial in healthy volunteers

**DOI:** 10.1186/1472-6882-14-351

**Published:** 2014-09-23

**Authors:** Vickram Beejmohun, Marie Peytavy-Izard, Cyril Mignon, Delphine Muscente-Paque, Xavier Deplanque, Christophe Ripoll, Nicolas Chapal

**Affiliations:** Dialpha SAS, Parc Agropolis 2, 2196 Boulevard de la Lironde, Montferrier sur Lez, 34980 France; Naturalpha SAS, Parc Eurasanté, 885 Avenue Eugène Avinée, Loos, 59120 France

**Keywords:** Cinnamon extract, Postprandial glycemia, Glycemic index, Alpha-amylase inhibitor, Starch digestion, Pre-diabetes, Metabolic syndrome

## Abstract

**Background:**

Postprandial hyperglycemia is a known risk factor for the development of several health disorders including type 2 diabetes, obesity, oxidative stress, and cardiovascular diseases. One encouraging approach for a better control of postprandial glycemia is to reduce carbohydrate digestion. Cinnamon extracts have been known for managing blood glucose. However, their effects on inhibiting digestion of carbohydrate have been poorly analyzed to date. The aim of this study was to investigate the acute effect of a specific Ceylon cinnamon hydro-alcoholic extract (CCE) on carbohydrate digestion and post-meal blood glucose reduction.

**Methods:**

*In vitro* enzymatic assays and *in vivo* starch tolerance tests in rats were designed as preclinical assays. Then, a randomized, double-blind, placebo-controlled, cross-over clinical trial was conducted in 18 healthy female and male volunteers. Following the intake of 1 g of CCE, the subjects ate a standardized meal. Blood samples were collected during the 2 hours following the meal to measure glucose and insulin concentrations. Areas under the curves were calculated and statistical differences between the CCE and placebo groups were analyzed using the Mann Whitney-Wilcoxon test.

**Results:**

CCE has demonstrated in the *in vitro* study that it inhibited pancreatic alpha-amylase activity with an IC50 of 25 μg/mL. In the *in vivo* study, CCE was shown to acutely reduce the glycemic response to starch in a dose-dependent manner in rats. This effect was significant from the dose of 12.5 mg/kg of body weight. In both, the *in vitro* and *in vivo* studies, the hydro-alcoholic extract has shown to be more efficacious than the aqueous extract. In the human clinical trial, 1 g of CCE lowered the area under the curve of glycemia between 0 and 120 min by 14.8% (P = 0.15) and between 0 and 60 min by 21.2% (P < 0.05) compared to the placebo. This effect occurred without stimulating insulin secretion. No adverse effects were reported.

**Conclusion:**

These results suggest that Ceylon cinnamon hydro-alcoholic extract (CCE) may provide a natural and safe solution for the reduction of postprandial hyperglycemia and therefore help to reduce the risks of developing metabolic disorders.

**Trial registration:**

ClinicalTrials.gov NCT02074423 (26/02/2014)

## Background

A large and growing body of evidence indicates that postprandial hyperglycemia is a contributing factor for the development of several health related disorders [1, 2]. Firstly, postprandial hyperglycemia has been shown to begin prior to type 2 diabetes occurring [3–5] and its control is essential for achieving recommended glycosylated hemoglobin levels (representing mean glycemia over a prolonged period of time) since control of fasting hyperglycemia only is necessary but usually insufficient [4]. The human and economic cost of diabetes could be significantly reduced through prevention, and the control of postprandial glycemia is a good area to target for this purpose. Secondly, several studies suggest that low glycemic index diets, inducing lower postprandial glycemic responses, improve lipid profiles and body weight gain patterns [6–9]. These diets decrease total fat mass [7, 8], total cholesterol and LDL cholesterol, thus improving plasma lipid profiles in healthy subjects [6, 7, 9]. Thirdly, a causal relationship has been demonstrated between postprandial hyperglycemia and oxidative stress [10], carotid intima-media thickness and endothelial dysfunction [11], and increased the risk of cardiovascular diseases occurring [12, 13]. Moreover, results from the Baltimore Longitudinal Study on aging showed that impaired glucose tolerance (IGT), by itself, increases the risk of cardiovascular diseases occurring [14]. Accordingly, each of these studies provide relevance for reducing postprandial hyperglycemia in order to prevent several metabolic disorders.

One interesting approach for limiting postprandial hyperglycemia is to reduce or slow down dietary carbohydrate digestion. Inhibiting the enzymes involved, such as the α-amylase and α-glucosidase enzymes, is a powerful therapeutic target for managing the postprandial glycemic response. Also, α-glucosidase inhibitors are one of the anti-diabetic drug families, of which Acarbose is the most well-known. These medications have a very strong effect and are suitable for treating type 2 diabetes [15] but also induce gastrointestinal side effects that limit their use in a preventive approach [16]. Consequently, several scientists are researching and developing nutritional strategies to finely regulate postprandial glycemia, without inducing adverse events in the gastric tract [17].

Cinnamon, one of the oldest spices known world-wide, is obtained from the inner bark of different tree species of the genus *Cinnamomum*. Chinese cinnamon (*Cinnamomum cassia* or *Cinnamomum aromaticum*), coming from China and Southern and Eastern Asia, is more widely used, notably in food, but it contains high level of coumarin, a potentially harmful molecule [18], unlike Ceylon cinnamon (*Cinnamomum zeylanicum* or *Cinnamomum verum*), coming from Sri Lanka and Madagascar, which contains only traces of coumarin [19]. Cinnamon has been extensively studied for the regulation of blood glucose. Several clinical trials have evaluated the chronic effects of cinnamon [20, 21] or cinnamon extracts [22, 23] on subjects suffering from type 2 diabetes. However, the results of several meta-analyses remain controversial, some indicating that cinnamon causes a significant decrease in fasting blood glucose [24, 25] where another reports that cinnamon does not improve fasting blood glucose, HbA1c or lipid parameters in type 2 diabetic subjects [26].

Regarding the acute effect of cinnamon on postprandial glycemia, the available literature is quite puzzling. *In vivo*, a methanol extract of Ceylon cinnamon reduced the glycemic response to maltose and sucrose in normal and diabetic rats but had no effect on glucose loading, thus suggesting a specific effect of this extract on disaccharide digestion through the inhibition of the α-glucosidase enzyme [27]. Some clinical studies have evaluated the acute effects of ground cinnamon bark on postprandial plasma glucose. Wickenberg *et al.* did not observe any significant effect with 6 g of Ceylon cinnamon during a standard oral glucose tolerance test (OGTT) in subjects with IGT [28], and Markey *et al.* saw no effect of 3 g of Ceylon cinnamon in response to a high fat breakfast in healthy volunteers [29]. For Cassia species, Hlebowicz *et al.* did show that the ingestion of 6 g of Cassia cinnamon reduced the blood glucose response to a test meal [30] while 3 g did not [31]. Magistrelli *et al.* confirmed the acute effect of 6 g Cassia cinnamon on postprandial glycemia in both: normal-weight and obese adults [32].

As illustrated by all of the above, some published clinical studies have assessed the acute effect of raw cinnamon powder from different cinnamon species on postprandial glycemia. However, to our knowledge, the acute effect of cinnamon extracts has not been clinically researched. As a result, we have identified a specific hydro-alcoholic Ceylon cinnamon extract. This publication presents the effects that the extract has on the pancreatic α-amylase activity and on the glucose response to starch in rodents as well as humans as tested in a randomized, placebo-controlled, cross-over clinical trial in healthy subjects.

## Methods

### Cinnamon extracts

The Ceylon cinnamon extract (CCE) tested in this study is manufactured by Dialpha (commercially available under the MealShape trademark). It is a hydro-alcoholic extract of Ceylon cinnamon bark (10:1), also called true Cinnamon, *Cinnamomum zeylanicum*, or *Cinnamomum verum*. The dried and milled cinnamon barks were extracted with a 50:50 water-ethanol solution during a 2 hour period. The extraction mixture was filtrated in order to remove any remaining solids, then concentrated under reduced pressure through evaporation of the ethanol and most of the water, before finally being dried in a vacuum drier to obtain a fine brown powder: CCE.

CCE is standardized at a minimum of 40% of polyphenols, CCE’s main active compounds. These polyphenols are mainly constituted of procyanidin oligomers which are composed of catechin and epicatechin monomers. The total polyphenol content was measured by a colorimetric method using a Folin-Ciocalteu reagent as per the ISO14502-1 method.

In order to compare the potential impact of the extraction solvent on the acute reduction of the starch glycemic response, an aqueous cinnamon extract was also produced from the same Ceylon cinnamon raw material as CCE, using the same extraction process.

### *In vitro*inhibition of the pancreatic α-amylase enzyme activity

Inhibition of α-amylase activity was assayed using the enzymatic assay and reagents proposed by Sigma Aldrich. Briefly, the pre-incubation mix was composed of 1.5 mL of buffer (50 mmol/L sodium phosphate, 50 mmol/L sodium chloride, 0.5 mmol/L calcium chloride, pH 6.9), 0.4 mL of the solutions to be tested containing the inhibitors at different concentrations, and 0.1 mL of the enzymatic solution consisting of 100 U/mL of α-amylase from porcine pancreas (Sigma Aldrich). It was pre-incubated for 30 min at 25°C. Then, 0.5 mL of this pre-mix was incubated with 0.5 mL of a substrate composed of 1% (w:v) gelatinized potato starch (Sigma Aldrich) in 20 mmol/L sodium phosphate buffer and 6.7 mmol/L sodium chloride (pH 6.9) for 20 min at 25°C. The reaction was stopped by the addition of 0.5 mL of a solution composed of sodium potassium tartrate and 96 mmol/L 3,5-dinitrosalicylic acid, then by boiling for 15 min, then by cooling on ice. The absorbance was read at 540 nm. The assays were conducted in triplicates. Acarbose (Sigma Aldrich) was used as a positive control.

### Acute starch tolerance test in the rat

Five-week old male Wistar Han IGS rats weighing between 50 and 75 g were purchased from the Charles Rivers Laboratories. Two rats were housed per cage. Filtered tap water and regular animal non-purified diet comprised of 51.7% carbohydrates, 21.4% proteins, and 5.1% lipids (diet A03, SAFE) were supplied *ad libitum*. The animal room environment was controlled with a temperature of 22 ± 2°C and day/night cycles of 12 hours light, 12 hours dark (19:00–7:00). Animals were allowed to acclimate to the laboratory environment for 10 days, weighed 3 times per week, and randomly assigned to the different study groups (8 to 20 animals per group depending on the study, as indicated in the figure legends). The protocols were approved by the following ethics committee: “*Comité Régional d’Ethique sur l’Expérimentation Animale*”, Pharmacology Laboratory, Pharmacy University, 15 Avenue Charles Flahault, 34093 Montpellier, France. Starch tolerance tests (STT) were conducted in animals which had fasted overnight through the acute administration by oral gavage of a 7.5% wheat starch solution at 1.5 g/kg or 20 mL/kg of body weight containing the products to be tested. The control group was administered starch only. The actual volume administered to each rat was calculated and adjusted based on the most recent body weight of each animal. Blood samples were collected via the tail vein before and 15, 30, 60, 90, and 120 min after starch administration. One drop of blood was used for the glucose determination using a hand-held glucometer (OneTouch Ultra 2, LifeScan) and, when insulin was measured, 50 μL of blood were withdrawn using heparin as an anticoagulant for the plasma preparation. Insulin concentrations were determined using an ELISA assay (Ultrasensitive Mouse Insulin ELISA, Mercodia). At the end of the studies, animals were euthanized by CO2 inhalation.

Three studies were performed, all following this protocol and referred to in the following sections of the Results section of this publication: effect of CCE at 50 mg/kg of body weight on blood glucose and insulin response, dose-effect of CCE at 6.25, 12.5, 25, 50, and 100 mg/kg of body weight on blood glucose response and differences of effects between hydro-alcoholic (CCE), and aqueous extracts at 50 mg/kg of body weight.

### Human clinical trial

This clinical study was conducted by Naturalpha, a Contract Research Organization (CRO), within their facilities of the Clinic Nutrition Center, Hôpital Saint Vincent de Paul, Lille, France. It consisted of a monocentric, randomized, double-blind, placebo-controlled, crossover clinical trial which was approved by the National Agency for Medicines and Health Products Safety (ANSM, 143/147 boulevard Anatole France, 93285 Saint-Denis, France) and the Ethics Committee (CPP Nord-Ouest III, CHU, avenue Côte de Nacre, 14033 Caen, France).

The primary endpoint was the area under the curve (AUC) 0–120 min of glycemia compared between CCE and placebo after the absorption of a standard meal. The secondary endpoints were the AUC 0–60 min of glycemia, the AUC 0–120 and 0–60 min of insulinemia, maximal glucose and insulin concentration, glycemia and insulinemia values at each point in time. The occurrence of adverse events (AE) was recorded during the entire study. AE have been either spontaneously reported by the subjects, collected through Investigator questioning, clinical examination or laboratory testing, or from Investigator reviews of the subject’s diary. The intensity of all AE was recorded on the Case Report Form as ‘mild’, ‘moderate’ or ‘severe’.

Before participating in the study, each volunteer signed an informed consent form. Inclusion criteria included age between 18 and 45, BMI between 18.5 (limit included) and 25 kg/m^2^ (limit excluded), good physical condition, and stable body weight within the last 3 months prior to screening (<5% variation). Exclusion criteria included fasting capillary blood glucose levels > 110 mg/dL, history of diabetes, smokers, intake of dietary supplements or drugs having an effect on glycemia and insulinemia. The subjects were required to maintain the same lifestyle throughout the study and to eat the same meal at dinner the evening before the two tests. The subjects eligible for the study in terms of inclusion and non-inclusion criteria had an OGTT at the screening visit (V1) in order to exclude subjects with atypical glycemic responses so as to have a homogeneous healthy population with a minimal glycemic response variation. After an overnight fast of at least 10 hours, subjects were given 50 g of glucose in 250 mL of water to drink within a 3 minute timeframe. Capillary blood samples were collected before and 15, 30, 45, 60, 90, and 120 min after the glucose load. Subjects with fasting capillary blood glucose levels > 110 mg/dL and/or 2 hours postprandial capillary blood glucose levels > 140 mg/dL were excluded from the study as well as subjects with AUC < Mean AUC ± 2 SD or AUC > Mean AUC ± 2 SD.

Every selected subject came to the clinical center after one night of fasting to complete two study visits (V2 and V3) at least 72 hours apart. Randomization and product assignment occurred during V2. It was performed using an allocation list prepared by a biostatistics company (Biostatem, France). The subjects were assigned in a random order to receive one of the following combinations: CCE, then placebo or placebo, then CCE. With this cross-over design, all subjects received both: the test product and the placebo, in a random order. The allocation list was used to label the study products accordingly. The study products were packaged and labeled by the Promoter (Dialpha, France), with the packaging and labeling enabling unconditional double blind administration by the individuals conducting the trial (investigator and staff; Naturalpha, France) or the subjects of the study.

The test product was delivered as two 500 mg capsules of pure CCE. The matching placebo was provided in two 500 mg capsules, visually identical to the CCE and containing a mix of 20% microcrystalline cellulose and 80% di-calcium phosphate. The test meal consisted of 103 g of white bread containing 52.2% of carbohydrates (of which 3.6% of sugars), 7.4% of proteins, 0.1% of lipids, and 3.3% of fibers (the bread thus corresponding to 50 g of complex carbohydrates, i.e. carbohydrates equivalent to three or more sugars). One drop of capillary blood was used for glucose determination using a hand-held glucometer (Accu-Check Performa, Roche). A catheter was placed in the antecubital vein of the subject’s arm to collect venous blood in dried tubes for serum preparation for insulin measurement by ELISA (Unicel DXi, Beckman). Capillary blood samples were collected at -35 minutes prior to meal absorption to check the fasting status (i.e. blood glucose levels ≤ 110 mg/dL). The capsules (test product or placebo) were consumed with 125 mL of water at -30 minutes. At 0 minutes, the test meal was served with 250 mL of water for consumption within a period of 8 minutes. Capillary and venous blood samples were collected at -10, -5, 15, 30, 45, 60, 90, and 120 minutes. The averages of plasma glucose levels at -10 and -5 minutes were used as the fasting (time “zero”) glycemia value, whereas insulin level at time -5 min was used as the fasting (time “zero”) insulinemia value.

The study was regularly monitored to ensure execution in accordance with the protocol, Good Clinical Practice (GCP), and other applicable regulations. The person in charge of monitoring reviewed and compared the Case Report Form (CRF) entries with original source data in order to check protocol adherence and to detect any data inconsistency or discrepancy.

### Statistical analyses

For the rat studies, statistical analyses were conducted using Microsoft Excel software. Student’s t-test (two-sample equal variance with a two-tailed distribution) was executed as indicated in the legends of the figures. Values of P < 0.05 were considered statistically significant when comparing the control group to the treated group.

For the clinical trial, the required sample population size was calculated considering a power of 80%, a standard deviation of 25%, an expected difference between placebo and CCE (based on *in vivo* CCE preclinical data) of 25%, and using Student’s t table. According to these hypotheses, 18 subjects had to be enrolled in the trial. Statistical analyses were performed on ITT (Intention To Treat) and PP (Per Protocol) populations using SAS® software version 9.3. Since we were evaluating the ability of an active extract to reduce the glycemic and insulinic responses to white bread, we decided to measure the “net incremental AUC”. The Net incremental AUC takes into account the negative increments of the curve. The trapezoid rule is applied for all increments, whether positive and negative. All tests were two-sided and significance was declared at a 0.05 threshold. An analysis of variance was performed on the AUC by testing the subject, period, and product effects. Global product effect was tested and, subsequently, product comparisons were performed on CCE compared to Placebo. The comparison tests followed the intra-individual design using a paired Student’s t-test or a non-parametric method in case of non-normality: the Koch method [33]. The difference between the two products was tested with the Mann Whitney-Wilcoxon test, taking into account the period effect. First, the equality of residual effects was tested on the sum of the two periods, then in case of no difference, the equality of direct effect between CCE and Placebo was tested using the difference of the two periods. The period effect could be tested using the same test on the crossover difference.

## Results

### Inhibition of the pancreatic α-amylase enzyme activity by CCE

**Figure 1:**
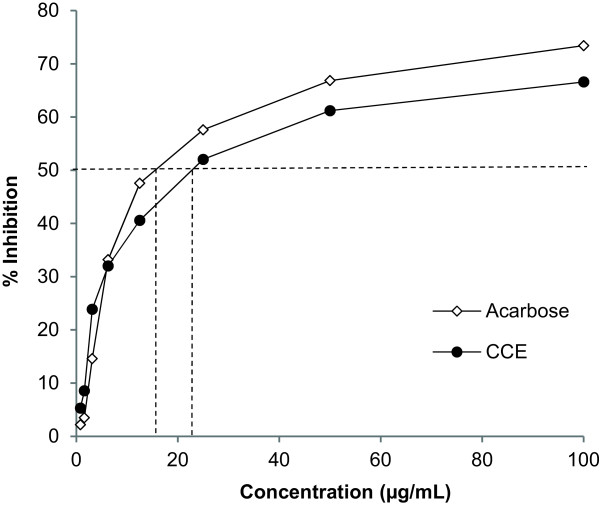
**Alpha-amylase inhibitory effect of CCE.** Values represent mean of triplicate measures.

CCE was tested for its ability to inhibit the activity of the α-amylase from the porcine pancreas. Acarbose, a well-known drug that inhibits α-glucosidase and α-amylase activity, was used as positive control. Both CCE and Acarbose inhibited mammalian α-amylase activity, with an IC50 of 25 and 18 μg/mL respectively (Figure 1).

### Effect of CCE on blood glucose and insulin response to starch in the rat

**Figure 2:**
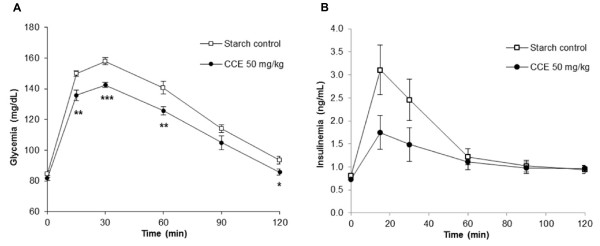
**Effect of CCE on blood glucose and insulin response during a STT in rats.** Acute effect of CCE at the dose of 50 mg/kg of body weight on blood glucose response **(A)** and on insulin response **(B)** during STT in normal rats. Values represent mean ± SEM, n = 8. Time points different from control, Student’s t-test *P < 0.05; **P < 0.01; ***P < 0.001.

The administration of 1.5 g/kg of body weight of starch in rats fasting overnight induced a quick and significant rise in glycemia, i.e. an increase of 73.5 ± 2.8 mg/dL above the pre-STT value (T0) 30 min after the starch load (Figure 2A). When added to starch, CCE at 50 mg/kg of body weight reduced the glycemic response by 20.4% compared to starch alone (AUC 0–120, P < 0.05). This effect is particularly significant during the peak of glycemia at 30 min (Student’s t-test P < 0.001; Figure 2A).

Without the CCE and during the same lapse of time, following the rise of glycemia-induced insulin secretion by the pancreas, the blood insulin level quickly rose reaching 2.3 ± 0.5 ng/mL above pre-STT value at the peak, 15 min after starch load (Figure 2B). With CCE, the insulin peak (AUC 0–120 min) was blunted by 40.6%, reaching 1.0 ± 0.4 ng/mL above pre-STT value at 15 min. This effect on insulin is statistically significant for the AUC 0–60 min (P < 0.05) but not for the AUC 0–120 min (P = 0.13).

### Dose-effect of CCE on blood glucose response to starch in the rat

**Figure 3:**
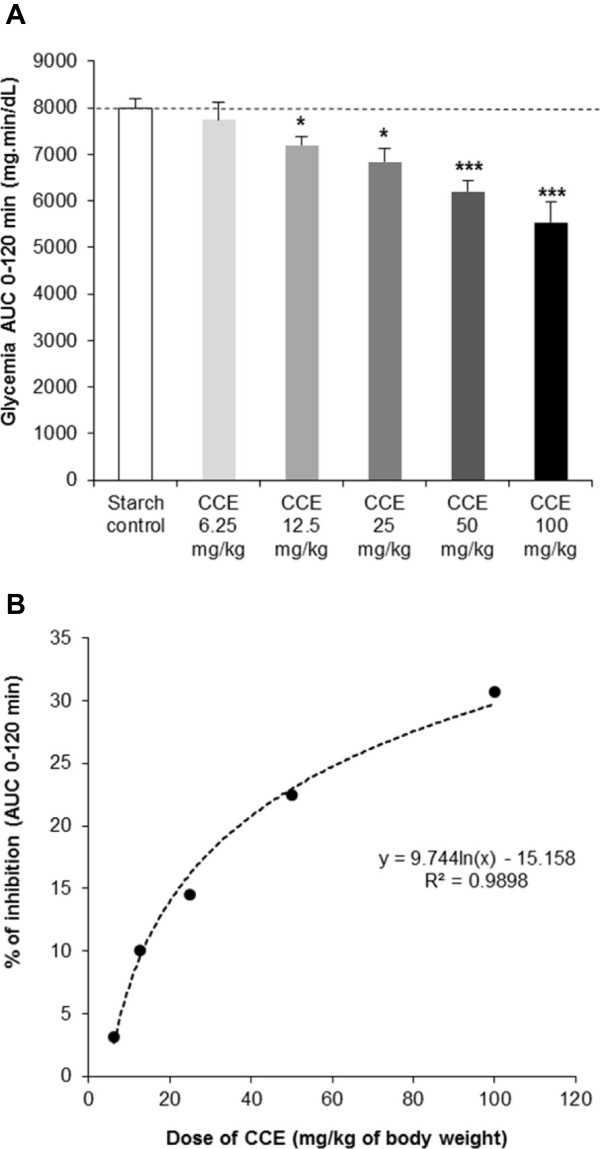
**Dose-effect of CCE on blood glucose during a STT in rat.** Acute dose-effect of CCE on blood glucose response during STT in normal rats, **(A)** AUC 0–120 min, **(B)** Logarithmic regression of CCE dose-effect. Values represent mean ± SEM, n = 8. AUC different from control, Student’s t-test *P < 0.05; ***P < 0.001.

The dose–response of CCE was evaluated on blood glucose levels during STT in normal fasting rats. CCE reduced the glycemic response to starch in a dose-dependent manner (Figure 3A). Compared to the control (starch alone), the AUC between 0 and 120 min was reduced by 3.2%, 10.1%, 14.5%, 22.5%, and 30.7% with CCE doses of 6.25, 12.5, 25, 50, and 100 mg/kg of body weight, respectively. This effect is significant from 12.5 mg/kg of body weight (Student’s t-test P < 0.05). A regression analysis of the dose–response of CCE were performed. The best fit was obtained using logarithmic equation (R^2^ = 0.99; Figure 3B) suggesting that CCE effect on STT may reach a plateau at doses higher than 100 mg/kg of body weight.

### Differences of effects between hydro-alcoholic and aqueous extracts

**Figure 4:**
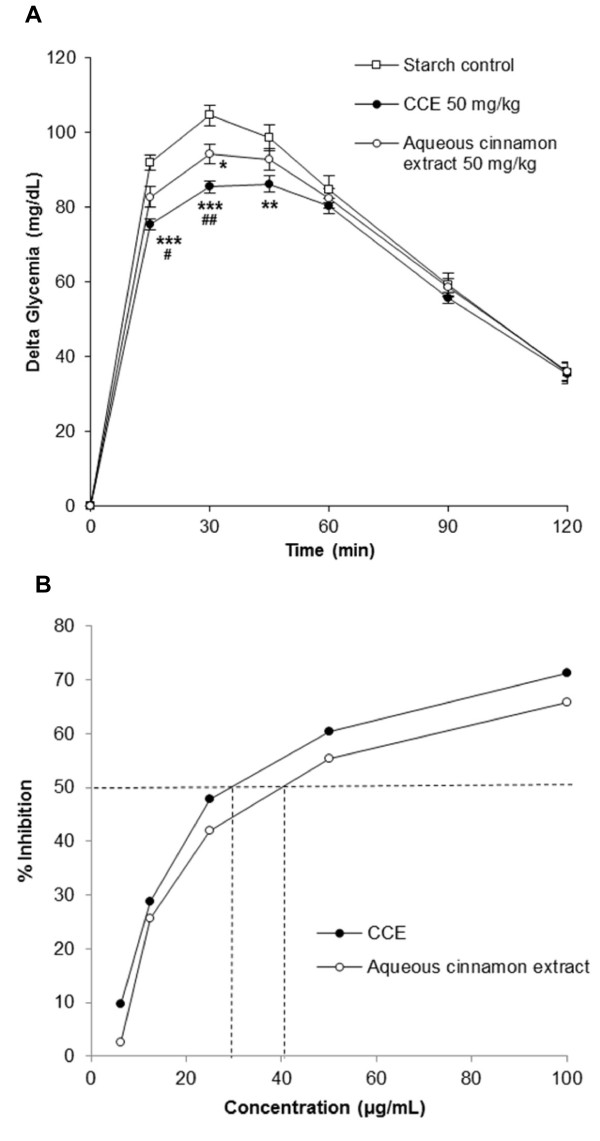
**Comparison of the effects of hydro-alcoholic and aqueous cinnamon extracts. (A)** Blood glucose response during STT in normal rats. Values represent mean ± SEM, n = 20. Student’s t-test: *P < 0.05; **P < 0.01; ***P < 0.001 different from control; ^#^P < 0.05; ^##^P < 0.01 different from aqueous cinnamon extract. **(B)** *In vitro* α-amylase inhibition. Values represent mean of triplicate measures.

The magnitude of effects on the reduction of glycemic response to starch in rats with the hydro-alcoholic extract (CCE; 14.9% reduction of AUC 0–60 min) is double compared with the aqueous extract (7.8% reduction of AUC 0–60 min) (Figure 4A). The effect of CCE is significantly higher at time point 15 min (P < 0.05), 30 min (P < 0.01), and for the AUC 0–60 min (P < 0.05) compared to the aqueous cinnamon extract.

The effects obtained on the α-amylase inhibition *in vitro* (Figure 4B) seem consistent with the effects obtained on the glycemic response in the rat: the hydro-alcoholic extract (CCE; IC50 = 30 μg/mL) presented a stronger inhibition than the aqueous extract (IC50 = 40 μg/mL).

### Effect of CCE in human clinical trial

**Figure 5:**
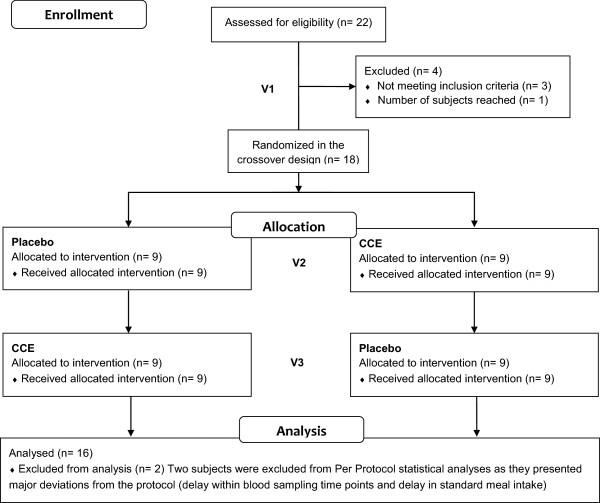
**Flow diagram of clinical study participants.**

Twenty-two subjects were screened for this study. Three subjects were declared non-eligible (blood glucose levels or BMI not compliant with inclusion criteria) and one subject was not included because the total number of subjects required for the study was reached. In total, 18 randomized subjects were included in the crossover study (Table 1). All 18 selected subjects completed the study. Compliance with the treatment reached 100% and no side effects were reported during the study. Two subjects were excluded from Per Protocol statistical analyses as they presented major deviations from the protocol (delay within blood sampling time points and delay in standard meal intake; the CONSORT flow diagram of participants is described in Figure 5). Sequence effect was investigated to compare “Product then Placebo” and “Placebo then Product” sequences. No statistical difference between sequences was observed.

**Figure 6:**
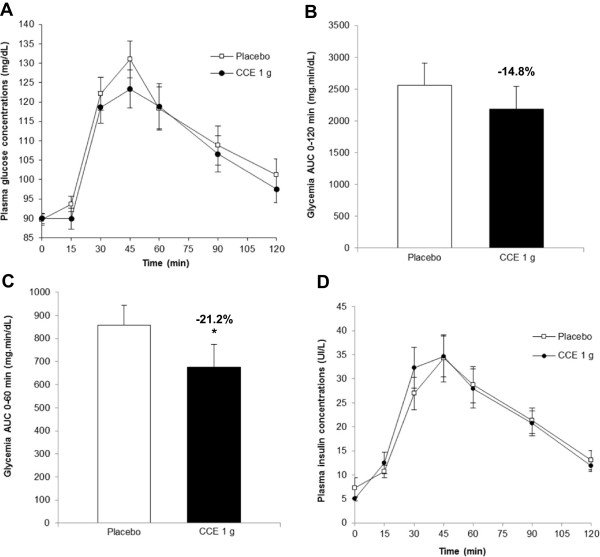
**Effect of 1 g CCE on blood glucose and insulin response after a standard meal in humans. (A)** Blood glucose concentration over 120 min, **(B)** Glycaemia AUC 0–120 min, **(C)** Glycemia AUC 0–60 min, and **(D)** Insulin concentrations over 120 min. Values represent mean ± SEM, n = 16.% of inhibition of test meal glycemic response is indicated. CCE different from placebo, *P < 0.05.

The intake of 1 g of CCE 30 minutes before the test meal reduced the glycemic response to this test meal compared to placebo (Figure 6A). CCE reduced the AUC 0–120 min by 14.8%, although a significant difference was not reached (P = 0.15; Figure 6B). However, a significant difference was observed at the peak of glycemia corresponding to the AUC 0–60 min. In these first 60 min, the consumption of CCE was associated with a 21.2% reduction of blood glucose compared to placebo (P < 0.05; Figure 6C). At the same time, no significant difference was observed between groups regarding all the other measured parameters, notably insulin response (Figure 6D). These results suggest that CCE ingestion prior to a standard meal helps limit the glucose response in the 60 minutes following the meal absorption without affecting insulin response.

**Table 1:** **Baseline characteristics of the healthy volunteers**

*n*	18
Age (years)	29.9 ± 1.8
Gender	11 male/7 female
BMI (kg/m^2^)	21.4 ± 0.4
Heart rate (bpm)	67.8 ± 2.4
Systolic blood pressure (mmHg)	127.3 ± 2.7
Diastolic blood pressure (mmHg)	73.2 ± 2.4

Cross-over design. Values represent mean ± SEM.

## Discussion

This study investigated for the first time the acute effect of a Ceylon cinnamon water-ethanol extract on blood glucose response to starch.

*In vivo* data provide evidence of the effect of CCE on the reduction of postprandial glycemia following starch administration in normal rats. The effect increased gradually with the dose and is significant from 12.5 mg/kg of body weight. Interestingly, at the same time, CCE also tended to lower insulin response to starch, showing that the effect of the extract is not due to a stimulation of insulin secretion. Next to that, other experiments that we have conducted in rats showed that if starch is being replaced with glucose, maltose or saccharose in the acute tolerance test, CCE has no effect on the glycemic response (data not shown). These results indicate that CCE acutely inhibits the digestion of starch *in vivo* but has no effect on the digestion of disaccharides (maltose and saccharose), nor on the passage of glucose from the intestine to the blood stream. Also, CCE showed no acute effect on glucose tolerance, i.e., the capacity of the body to manage glycemia after the passage of glucose into the blood.

Taken together, these results support the fact that CCE seems to specifically inhibit α-amylases. One published study demonstrating the acute effect of a methanol Ceylon cinnamon extract at 300 mg/kg of body weight on the glycemic response to maltose and sucrose in rats suggested an effect on alpha-glucosidase [27]. We could not reproduce these results with CCE at the dose of 50 mg/kg of body weight, a dose which is clearly effective on starch response and which constitutes, in our point of view, a more physiological dose regarding further applications in humans.

The effects of CCE on postprandial blood glucose and insulin response were assessed in healthy male and female volunteers. Since the extract seems to act by inhibiting α-amylase enzymes, the first enzymes involved in carbohydrate digestion, and since CCE was formulated in capsules, we wanted to make sure that the capsules would dissolve and that the enzymes would be inhibited before the arrival of the test meal in the upper intestine. For this reason, we decided to administrate the capsules 30 minutes before the meal consumption. In doing so, 1 g of CCE reduced the post-meal glycemic response by ~ 20% and ~ 15% between 0 and 60 min and between 0 and 120 min post meal absorption respectively. This effect is statistically significant (P < 0.05) at the peak of glycemia between 0 and 60 min post meal absorption. In this trial, we addressed young healthy volunteers with normal glucose tolerance (IGT subjects were excluded at screening). Accordingly, in these subjects, glycemia is mainly increased between 0 and 60 min post meal absorption and returns to normal value between 60 and 120 min, almost linearly (Figure 6A). In these conditions, it is understandable that the effect of CCE is higher on the peak where the “therapeutic window” is larger. By increasing the number of subjects, our observed glycemic response reduction could possibly also have become statistically significant between 0 and 120 min. Another option could have been to select IGT subjects presenting an exacerbated glycemic response which remains elevated until 2 hours after the meal absorption.

No significant difference was observed in terms of insulin response between CCE and placebo, showing that the effect of CCE in reducing postprandial glycemia does not seem to be due to a stimulation of insulin secretion. This is consistent with the findings from the rat study.

Regulatory bodies consider that the improvement of post-prandial glycemia should not be accompanied by an increase in insulinemia which is in accordance with this clinical trial’s findings.

In conclusion, women and men who have taken CCE have reduced their glycemia following the absorption of the test meal, yet with no variation of their insulin demand.

Interestingly, as a side finding, this work allows us to compare the acute effect of CCE, an α-amylase inhibitor acting in the intestine, on the glycemic response to starch in rats and in humans. To our knowledge, this correlation is poorly described in existing literature. 1 g of CCE (~12.5 mg/kg of body weight for a man weighing 75 kg) resulted in the reduction of the glycemic response to starch in humans by 15 to 20%. Given that a dose of 25 to 50 mg/kg of body weight is needed to observe an equivalent reduction in the rat, it appears that the human equivalent dose of CCE or, by extension, a “CCE-like” α-amylase inhibitor, to the rat may be the dose effective in the rat divided by 2 to 4.

Here we tested the acute effect of a specific hydro-alcoholic Ceylon cinnamon extract on postprandial glycemia. This is a new discovered property of cinnamon extracts which are usually recognized for their chronic effect on glycemia. Other investigators have tested the effect of up to 6 g of raw cinnamon bark powder on the glycemic response in human clinical trials. No effect was observed with Ceylon cinnamon [28, 29] whereas *cassia* cinnamon presented significant effect in 2 studies [30, 32].

These interesting results bring us to another important consideration regarding the safety of cinnamons and their derived extracts with respect to their levels of coumarin, a potentially toxic molecule [18]. Ceylon cinnamon contains only traces of coumarin and the corresponding extract, even using ethanol as the extraction solvent, contains far less coumarin amounts than the tolerable daily intake established by the European Food Safety Authority (0.1 mg/day/kg of body weight; 7 mg/day for a person of 70 kg). The CCE coumarin contents is less than 200 mg/kg (i.e. 1 g of CCE/day contains less than 0.2 mg/day of coumarin). The coumarin content in the CCE batch used for the clinical trial was 112 mg/kg. For the cassia species however, coumarin levels are of concern [19]. Furthermore, since coumarin is more alcohol soluble than water soluble, an ethanol extract of this species would enrich the extract in coumarin, thus worsening coumarin related problem altogether. An aqueous extraction of the *Cinnamomum cassia* species would allow to reduce the level of coumarin contained in the raw material. However, according to the *in vitro* results on α-amylase inhibition and as evidenced by the *in vivo* results on starch tolerance tests in rats, CCE was significantly more efficient than an aqueous cinnamon extract.

Taken all together, these results show that a hydro-alcoholic Ceylon cinnamon extract appears to be an optimal solution in terms of coumarin content and efficacy as far as cinnamon extracts for use in the management of postprandial glycemia are concerned.

## Conclusions

In summary, this study demonstrates that a well-defined hydro-alcoholic Ceylon cinnamon bark extract reduces the glycemic response to starch in normal rats and healthy male and female subjects by inhibiting pancreatic α-amylase starch digestion. This extract may be of great interest with regards to the many recognized benefits associated with the reduction of postprandial hyperglycemia. These benefits namely include reducing the risk of developing type 2 diabetes in people with IGT, helping with the lipid profile management, helping with the control of fat mass, body weight, and oxidative stress, and finally helping reduce the risk for cardiovascular diseases occurring. Other human clinical trials will be required to confirm these results on postprandial glycemia and in order to directly assess the other health benefits.

## Abbreviations

CCE: Ceylon cinnamon hydro-alcoholic extract
HbA1c: Glycosylated hemoglobin
IGT: Impaired glucose tolerance
OGTT: Oral glucose tolerance test
STT: Starch tolerance test
AUC: area under the curve
